# Uptake of Intermittent Preventive Treatment and Its Associated Factors Among Pregnant Women in Cameroon: A Cross-Sectional Study

**DOI:** 10.7759/cureus.89187

**Published:** 2025-08-01

**Authors:** Eric T Defo, Armand T Tsapi, Martin T Fossi, Gaelle T Magne, Olivier Ethgen, Georges Nguefack-Tsague

**Affiliations:** 1 Public Health, University of Liege, Liege, BEL; 2 Public Health, Evangelic University of Cameroon, Bandjoun, CMR; 3 Medicine, University of Liege, Liege, BEL; 4 Public Health, Health Education and Development Association (HEADA) Cameroon, Yaoundé, CMR; 5 Public Health, University of Yaoundé 1, Yaounde, CMR

**Keywords:** antenatal care visits, cameroon, intermittent preventive treatment, malaria in pregnancy, sulfadoxine-pyrimethamine

## Abstract

Introduction: Malaria remains a major public health threat in sub-Saharan Africa. In Cameroon, where malaria is endemic, pregnant women are especially vulnerable due to reduced immunity and placental sequestration of infected erythrocytes. Intermittent preventive treatment in pregnancy (IPTp) with sulfadoxine-pyrimethamine (SP) is recommended by the World Health Organization (WHO) to prevent malaria-related complications. However, national coverage remains below the 80% target. This study aimed to assess IPTp uptake and its associated factors among pregnant women in five diverse regions of Cameroon.

Methods: A cross-sectional study was conducted between 2020 and 2022 in five health areas, using two-stage cluster sampling. A total of 259 pregnant women aged 15-49 were interviewed using a structured questionnaire. Data collected included sociodemographic characteristics, antenatal care (ANC) attendance, knowledge of malaria and IPTp, and prevention practices. Malaria infection was assessed using rapid diagnostic tests (RDTs). Logistic regression analysis was used to identify factors associated with the uptake of at least three doses of IPTp.

Results: Among participants, 62.55% (CI95%: 56.31% to 68.40%) had received at least three doses of IPTp. Age was a significant predictor: women aged between 25 and 30 years had higher odds of optimal uptake (adjusted odds ratio (aOR): 4.87; 95% CI: 2.15-11.51), and those over 30 had even greater odds (aOR: 7.93; 95% CI: 3.08-21.71) compared to those aged between 21 and 25 years. Marital status also influenced uptake: married women (aOR: 9.52; 95% CI: 4.29-23.27) and cohabiting women (aOR: 2.85; 95% CI: 1.50-5.56) were more likely to receive three doses than single women. Parity was associated with higher adherence, with primiparous (aOR: 8.25; 95% CI: 3.02-26.69) and multiparous women (aOR: 7.26; 95% CI: 2.17-27.74) more likely to complete IPTp than nulliparous women. ANC attendance was a key determinant: women with more than two visits were significantly more likely to complete IPTp (aOR: 2.41; 95% CI: 1.17-4.96). Good malaria knowledge (aOR: 1.99; 95% CI: 1.12-3.58) and good IPTp knowledge (aOR: 2.19; 95% CI: 1.10-4.38) were also positively associated. Testing positive for malaria was associated with lower IPTp adherence (aOR: 0.53; 95% CI: 0.31-0.92).

Conclusion: Despite improvements compared to national statistics, IPTp uptake remains below WHO recommendations. Key factors influencing adherence include age, marital status, parity, ANC frequency, and knowledge levels. Higher IPTp uptake was associated with lower malaria prevalence, which suggests but does not confirm a protective effect given the cross-sectional design. Public health strategies should focus on promoting early and regular ANC attendance, improving education on malaria prevention, and encouraging male involvement to boost IPTp coverage and improve maternal outcomes.

## Introduction

Malaria remains a major public health concern globally. In 2023, an estimated 263 million malaria cases were recorded worldwide, representing an increase of approximately 11 million cases compared to the 249 million reported in 2022 [1]. Pregnant women are among the most vulnerable to malaria due to decreased immunity during pregnancy (especially during the first and second pregnancies) and the sequestration of infected erythrocytes in the placenta [1]. This vulnerability contributes significantly to maternal and neonatal morbidity and mortality. An estimated 10,000 women and 200,000 infants die each year because of malaria in pregnancy; severe maternal anemia, prematurity, and low birth weight account for over half of these deaths [2].

Although malaria cases increased, the number of related deaths remained almost unchanged, with approximately 597,000 deaths reported [1]. The World Health Organization (WHO) African Region bears a disproportionately high share of the global malaria burden, accounting in 2023 for 94% of all malaria cases and 95% of malaria-related deaths worldwide. Children under five represented about 76% of all malaria deaths in the region [1]. The highest burden of malaria is borne by 11 countries, including Cameroon [1].

In Cameroon, the entire population is exposed to malaria, and for this reason, this disease is highly endemic. According to the National Surveillance Report, every year, Cameroon registers around six million cases of malaria, and health facilities record about 4,000 deaths, most of which occur in children below the age of five [3]. However, not all cases and deaths are recorded, and the WHO estimates that about 11,000 people die from malaria in Cameroon every year [3]. Around 30% of all outpatient visits to healthcare facilities are for malaria, making it a disease of importance in Cameroon [3].

Building on national burden trends from previous years (2022-2023), the most recent figures further illustrate the ongoing impact of the disease. In 2024, according to the Ministry of Health (MoH), data reported by healthcare facilities across the country indicated 2,964,132 confirmed malaria cases, representing a proportional morbidity of 26.9% [4]. Among these cases, 2,016 deaths were recorded, accounting for 8.2% of all reported deaths. These figures show a slight decrease compared to 2023, when 2,977,754 malaria cases were reported (proportional morbidity of 27.6%) along with 1,756 deaths (proportional mortality of 7.3%) [3].

Specifically among pregnant women, who remain a high-risk group for malaria, the proportional morbidity related to malaria in 2024 was 21.2%, with a mortality rate of 7.7% [4].

Malaria infection in pregnant women can induce maternal anemia, low birth-weight infant delivery (<2500 g or <5.5 pounds), premature delivery, or fetal loss [5]. To prevent malaria infection during pregnancy, the WHO recommends the use of intermittent preventive treatment in pregnancy (IPTp) with sulfadoxine-pyrimethamine (SP). This involves administering at least three doses of SP, starting as early as possible in the second trimester, with each dose given at least one month apart [1]. In practice, however, the timing and number of doses may vary depending on when pregnant women initiate antenatal care (ANC) and how regularly they attend follow-up visits, which is often influenced by individual health status and access to services in settings like Cameroon [1]. For this reason, the Cameroonian government has, since 2007, adopted the free administration of at least two doses of SP-based IPTp to all pregnant women during ANC visits [6]. Since 2012, the minimum number of doses has been increased to three following WHO recommendations [6]. However, IPTp observance by pregnant women remains relatively low. Indeed, according to Cameroon’s National Malaria Control Program (NMCP), only 51.8% of pregnant women had received at least three doses of IPTp in 2024 during ANC visits [3]. Hence, this study aimed to identify the sociodemographic, knowledge-related, and health system factors influencing IPTp uptake among pregnant women in five selected health areas across Cameroon in order to provide key insights for strengthening the effectiveness of this preventive strategy.

## Materials and methods

To achieve our objective, we conducted a cross-sectional household-based study. Given that malaria is endemic throughout the country, a purposive selection of five out of the 10 administrative regions was made, based on security, accessibility, and operational feasibility constraints. Within each selected region, a health district was then randomly selected using a simple random sampling method (random number generator), followed by the random selection of one health area per district. The final five health areas included in the study were Fiala-Foréké (Dschang District, West Region), Ahala 1 (Efoulan District, Centre Region), Kye-Ossi (Ambam District, South Region), Akwa 1 (Deido District, Littoral Region), and Garoua-Winde (Garoua 1 District, North Region).

To reach households within each selected health area, we used a two-stage cluster sampling approach based on enumeration areas (EAs) provided by the National Institute of Statistics. In the first stage, EAs were randomly selected from the national sampling frame using probability proportional to size. In the second stage, a fixed number of 22 households per EA was selected through systematic random sampling. A total of 64 EAs were chosen to ensure adequate geographic spread and sample size. Sampling weights were computed using the inverse of the selection probability at each stage to ensure weighted national estimates.

The sample size for the overall household survey was calculated using R software (version 4.1.2, R Foundation for Statistical Computing, Vienna, Austria), based on the total population of the five selected health areas (164,208 individuals). Using a malaria morbidity rate of 29.6% reported for Cameroon in 2021, a 5% margin of error, a design effect (DEFF) of two, and a non-response inflation factor of 1.11, the required effective sample size was 320 individuals per domain (rural and urban), totaling 1,280 individuals for the general survey [7]. This resulted in 1,421 households being visited across the 64 EAs, with an average of 22 households per EA, balancing operational capacity with statistical requirements.

To estimate the number of pregnant women likely to be captured, we used a pregnancy prevalence estimate of 3.69%, derived from the population data produced by the MoH’s Health Information Unit [8]. Applying this rate to the general population, we expected to identify approximately 52 households with pregnant women, sufficient to support the analysis of IPTp uptake.

Data collection was done in two phases (from November 2020 to July 2021, then from December 2021 to June 2022) due to logistical constraints related to covering the five selected regions. 

The questionnaire was structured to answer questions concerning the socio-demographic and economic characteristics, knowledge, and attitudes regarding malaria, and the IPTp uptake of respondents.

During our research, we also performed malaria rapid diagnostic tests (RDTs) by using the VIKIA Malaria antigen (Ag) *Plasmodium falciparum* (*P. falciparum*)/*Plasmodium species* (*P. Pan*) test (Biomerieux, France, with sensitivity and specificity of more than 93%, to determine malaria prevalence in our study population. Participants with a positive result were referred to one of the health facilities for management according to national treatment guidelines.

The sampling included any pregnant women living in one of the five health areas mentioned above during the study period, aged between 15 and 49 years (reproductive age), and who had given their informed consent to participate. 

The assessment of knowledge was based on six questions, which were used to generate knowledge scores ranging from 0 to 1. These scores allowed us to categorize the levels of knowledge as "Good," "Moderate," or "Bad."

To assess the physical condition of insecticide-treated nets (ITNs), we adhered to the guidelines established in 2011 by the World Health Organization Pesticide Evaluation Scheme (WHOPES), which provides a structured approach for physical integrity evaluation of ITNs by counting the number of holes and calculating the proportionate hole index (pHI) [9]. The process began with classifying hole sizes based on their approximate diameter, using reference points such as the thumb (0.5-2 cm), fist (2-10 cm), head (10-25 cm), or larger than the head (≥ 25 cm). Holes smaller than 0.5 cm in diameter were excluded from the analysis, as the pHI method assumes that mosquitoes cannot penetrate openings of this size [10]. The next step involved calculating the pHI by assigning weights to each hole according to its size [9].

The IPTp uptake was determined based on the number of IPT doses taken during pregnancy (less than three or three to more than three doses) [11].

The sample size calculation was performed using R software version 4.1.2 with the “samplingbook” package. For this estimation, we used the 2019 population size of women of reproductive age in each selected health area, the malaria prevalence among pregnant women reported by the NMCP (19.6 per 100 inhabitants), a 95% CI, and a precision of 0.05 [12]. A design effect of two and a non-response adjustment factor of 1.11 were also applied, consistent with the parameters used for the overall household sampling. No finite population correction was applied.

Before analysis, the data were encoded in a Microsoft Excel file version 2016 (Microsoft Corp., Redmond, WA) and exported to R software for analysis.

Quantitative variables following a normal distribution were presented as mean ± standard deviation, and presented as median (interquartile range) otherwise. Frequencies and percentages (%) were used to describe qualitative variables. The factors associated with IPTp uptake were determined using the binary logistic regression method (both univariate and multivariate regression were performed). P-values less than 5% were considered statistically significant.

## Results

Sociodemographic characteristics of the study population

During our study, data were collected from a total of 259 households, each of which included one pregnant woman meeting the inclusion criteria, yielding a final sample size of 259 individual participants. A household was defined as a group of individuals sharing the same dwelling and meals, and for the purpose of this study, only one eligible pregnant woman per household was surveyed.

From a sociodemographic perspective, the majority of participants (62.93%, or 163/259) were between the ages of 25 and 30. Most were primigravida (77.22%, or 200/259), indicating women experiencing their first pregnancy; this information was self-reported during the interview. In terms of household income, 69.11% (179/259) of participants reported a monthly household income of 100,000 Central African CFA Francs (XAF) or less (approximately 152.45 Euros). While this threshold is not an official poverty line, it reflects a commonly cited benchmark for basic subsistence income in Cameroon and helps contextualize financial barriers to accessing care.

Geographical access to health facilities was also a concern: 32.43% (84/259) of the pregnant women lived more than 10 kilometers away from the nearest health facility. Additionally, 11.2% (29/259) reported having at least one child under the age of five living in their household (Table 1).

**Table 1:** Characteristics of respondents n: number of participants; (%): percentage

Variable	n (%)
Sociodemographic variables	
Age (years)	
21 to 25	38 (14.67)
25 to 30	162 (62.93)
More than 30	58 (22.39)
Distance between the respondent’s house and the health facility	
Less than 2 km	61 (23.55)
Between 2 and 10 km	114 (44.02)
Between 10 and 20 km	55 (21.24)
More than 20 km	29 (11.20)
Having children under the age of five in the household	
No	230 (88.8)
Yes	29 (11.2)
Income (Central African CFA Francs (XAF))	
0 - 99,999 (0 - 152.45 Euro)	179 (69.11)
100,000 - 199,999 (152.45 - 304.90 Euro)	22 (8.49)
200,000 - 300,000 (304.90 - 457.35 Euro)	47 (18.15)
More than 300,000 (More than 457.35 Euro)	11 (4.25)
Marital status	
In a relationship	98 (37.84)
Cohabiting	91 (35.14)
Maried	70 (27.03)
Level of education	
Primary	56 (21.62)
Secondary	46 (17.76)
Higher education (Non-university)	98 (37.84)
Higher education (University)	59 (22.78)
Region	
Centre	77 (29.73)
Littoral	42 (16.22)
North	51 (19.69)
South	24 (9.27)
West	65 (25.10)
Reproductive status and antenatal care (ANC) attendance variables	
Number of ANC visits	
One	47 (18.15)
Two	96 (37.07)
More than two	116 (44.79)
Parity	
Nulliparous	25 (9.65)
Primiparous	200 (77.22)
Multiparous	34 (13.13)
Malaria knowledge and prevention practices variables	
Knowledge about malaria	
Bad	0 (0)
Moderate	91 (35.14)
Good	168 (64.86)
Knowledge about Intermittent preventive treatment in pregnancy (IPTp)	
Bad	61 (23.55)
Moderate	73 (28.19)
Good	125 (48.26)
Number of mosquito nets in the household	
None	70 (27.03)
One	167 (64.48)
More than one	22 (8.49)
Proportionate hole index (pHI) of identified mosquito nets	
Acceptable	44 (23.28)
Degraded	66 (34.92)
Good	79 (41.80)
Malaria history and treatment-seeking behavior variables	
Had malaria in the past month	
No	171 (66.02)
Yes	88 (33.98)
Sought consultation during the malaria episode	
No consultation	35 (14.89)
Consultation at a conventional hospital	130 (55.32)
Consultation with a traditional healer	70 (29.79)
Treatment received during the malaria episode	
Conventional treatment	120 (49.59)
Traditional treatment	70 (28.93)
Both	52 (21.49)
Medication purchase	
By prescription	91 (37.60)
By self-medication	42 (17.36)
Both	109 (45.04)
Rapid diagnostic test (RDT)	
Negative	166 (64.09)
Positive	93 (35.91)
IPTp uptake	
< 3 doses	97 (37.45)
3 + doses	162 (62.55)

Regarding malaria prevention practices, 64.86% (168/259) of the participants demonstrated good knowledge of malaria, while 48.26% (125/259) had good knowledge of IPTp. Knowledge was assessed using a structured questionnaire composed of five questions; participants scoring at least three correct answers were classified as having “good knowledge.” Despite this, 27.03% (70/259) of participants reported not owning a mosquito net, and among households with at least one net, 34.92% (66/189) stated that their nets were in poor condition (Table 1).

Finally, regarding IPTp uptake, 62.55% (162/259) of the participants reported having received three or more doses of IPTp (Table 1).

Association between sociodemographic variables and IPTp uptake

Regarding sociodemographic variables, two factors were significantly associated with higher odds of receiving three or more doses of IPTp: age and marital status. In the logistic regression model, women aged between 21 and 25 years served as the reference group for age, while women “in a relationship” (not married or cohabiting) were the reference category for marital status.

Compared to women aged betwen 21 and 25 years, those aged between 25 and 30 years had significantly higher odds of receiving at least three doses of IPTp (adjusted odds ratio (aOR) = 4.87; 95% CI: 2.15-11.51; p = 0.0002), and women over 30 had even higher odds (aOR = 7.93; 95% CI: 3.08-21.71; p < 0.0001). Regarding marital status, married women had 9.52 times higher odds (aOR = 9.52; 95% CI: 4.29-23.27; p < 0.0001), and those cohabiting had 2.85 times higher odds (aOR = 2.85; 95% CI: 1.50-5.56; p = 0.0016) compared to women in a relationship (Table 2).

**Table 2:** Association between sociodemographic variables and intermittent preventive treatment in pregnancy (IPTp) uptake cOR: crude odds ratios; aOR: adjusted odds fatios; *P-value <0.05

Variable	cOR (IC95%)	P-value	Global P-value	aOR (IC95%)	P-value	Global p-value
Age (years)						
21- 25	1		0.0002	1		
26-30	3.58 (1.73-7.72)	0.0007*		4.87 (2.15-11.51)	0.0002*	< 0.0001*
31 or more	5.51 (2.31-13.84)	0.0001*		7.93 (3.08-21.71)	< 0.0001*	
Distance between the respondent’s house and health facility						
2 or less	1		0.5426	-	-	-
3-10	0.77 (0.40-1.47)	0.4375				
11-20	1.15 (0.54-2.52)	0.7057		-	-	-
21 or more	1.25 (0.50-3.32)	0.6391		-	-	-
Having children under five in the household						
No	1					
Yes	0.93 (0.55-1.58)	0.7775	0.7775	-	-	-
Income (Central African CFA Francs (XAF))						
0 - 99.999 (0 - 152.45 Euro)	1		0.2	-	-	-
100.000 - 199.999 (152.45 - 304.90 Euro)	0.49 (0.19-1.18)	0.1138				
200.000 - 300.000 (304.90 - 457.35 Euro)	1.03 (0.53-2.04)	0.9292		-	-	-
More than 300.000 (More than 457.35 Euro)	2.63 (0.65-17.58)	0.2253		-	-	-
Marital status						
Being in a relationship	1		< 0.0001*	1		< 0.0001*
Cohabiting	1.88 (1.06 - 3.38)	0.0322*		2.85 (1.50-5.56)	0.0016*	
Married	7.98 (3.71-18.85)	< 0.0001*		9.52(4.29-23.27)	< 0.0001*	
Level of education						
Primary	1		0.0977	-	-	-
Secondary	2.13 (0.93-5.06)	0.0802				
Higher education (Non-university)	0.96 (0.49-1.86)	0.9022		-	-	-
Higher education (University)	1.71 (0.80-3.71)	0.1708		-	-	-
Region						
Center	1		0.0619	-	-	-
Littoral	0.39 (0.18-0.85)	0.0026*				
North	1.05 (0.49-2.27)	0.8965		-	-	-
South	1.44 (0.53-4.38)	0.4899		-	-	-
West	0.68 (0.34-1.34)	0.2645		-	-	-

To ensure model validity, multicollinearity diagnostics were performed using the variance inflation factor (VIF), and no concerning collinearity was detected. The model also demonstrated acceptable stability and convergence.

Association between reproductive status, ANC attendance variables, and IPTp uptake

Regarding ANC attendance, the reference category in the model was women who had only one ANC visit. Women who had more than two visits were significantly more likely to receive at least three doses of IPTp (aOR = 2.41; 95% CI = 1.17-4.96; p = 0.0158). Although women with exactly two ANC visits also showed increased odds (aOR = 1.67; 95% CI = 0.79-3.56), this association was not statistically significant, and the relatively wide CI may reflect limited statistical power or smaller subgroup size within this category.

As for parity, both primiparous and multiparous women had significantly higher odds of receiving three or more doses of IPTp compared to nulliparous women (reference category). Primiparous women were 8.25 times more likely (aOR = 8.25; 95% CI = 3.02-26.69; p = 0.0001), and multiparous women 7.26 times more likely (aOR = 7.26; 95% CI = 2.17-27.74; p = 0.002), to complete the recommended IPTp schedule (Table 3).

**Table 3:** Association between reproductive status, ANC attendance variables, and intermittent preventive treatment in pregnancy (IPTp) uptake cOR: crude odds ratios; aOR: adjusted odds ratio; *P-value <0.05

Variable	cOR (IC95%)	P-value	Global P-value	aOR (IC95%)	P-value	Global P-value
Number of antenatal care (ANC) visits						
1	1		0.0116*	1		0.0523
2	1.08 (0.54-2.19)	0.82		1.67 (0.79-3.56)	0.1772	
3 or more	2.31 (1.14-4.69)	0.0196*		2.41 (1.17 -4.96)	0.0158*	
Parity						
Nulliparous	1		< 0.0001*	1		< 0.0001*
Primiparous	8.5 (3.27(26.46)	< 0.0001*		8.25 (3.02-26.69)	0.0001*	
Multiparous	6.46 (2.06-23.43)	0.0023*		7.26 (2.17-27.74)	0.002*	

Association between malaria knowledge, prevention practices variables, and IPTp uptake

The results presented in Table 4 indicate that malaria knowledge and IPTp knowledge were significantly associated with the likelihood of receiving three or more doses of IPTp, while mosquito net-related practices did not show a statistically significant effect.

**Table 4:** Association between malaria knowledge, prevention practices variables, and intermittent preventive treatment in pregnancy (IPTp) uptake cOR: crude odds ratios; aOR: adjusted odds ratio; *P-value <0.05

Variable	cOR (IC95%)	P-value	Global P-value	aOR (IC95%)	P-value	Global P-value
Knowledge about malaria						
Moderate	1			1		
Good	2.35 (1.39-3.98)	0.0014*	0.0014*	1.99 (1.12-3.58)	0.0197*	0.0198*
Knowledge about intermittent preventive treatment in pregnancy (IPTp)						
Bad	1		0.0021*	1		0.022*
Moderate	1.49 (0.76-2.98)	0.2492		1.98 (0.51-2.28)	0.834	
Good	2.95 (1.57-5.63)	0.0009*		2.19 (1.10-4.38)	0.0251*	
Number of mosquito nets in the household						
0	1		0.6198	-	-	-
1	0.75 (0.41-1.34)	0.3345				
2 or more	0.86 (0.32-2.41)	0.7616		-	-	-
Proportionate hole index (PHI) of identified mosquito nets						
Acceptable	1		0.4296	-	-	-
Degraded	0.69 (0.31-1.49)	0.3445				
Good	1.04 (0.47-2.23)	0.9187		-	-	-

For malaria knowledge, women with good knowledge were significantly more likely to receive at least three doses of IPTp compared to those with moderate knowledge (aOR = 1.99; 95% CI = 1.12-3.58; p = 0.0198). This model used moderate knowledge as the reference category to better capture the relative effect of strong malaria knowledge, although this differs from the coding used for IPTp knowledge and should be interpreted accordingly (Table 4).

Regarding IPTp knowledge, women with good knowledge had significantly higher odds of receiving three or more doses compared to those with limited knowledge (aOR = 2.19; 95% CI = 1.10-4.38). While statistically significant, the wide confidence interval suggests limited precision, and the result should be interpreted with caution. The association between moderate knowledge and IPTp uptake was not statistically significant (aOR = 1.98; 95% CI = 0.51-2.28), suggesting that only higher levels of IPTp knowledge may translate into behavioral uptake (Table 4).

In contrast, neither ownership of mosquito nets nor their physical condition (as assessed by the pHI) was significantly associated with IPTp uptake. This lack of association may reflect distinct behavioral determinants and health messaging surrounding IPTp and ITNs, despite both being core malaria prevention strategies. IPTp uptake depends heavily on interactions with the health system (e.g., ANC attendance), whereas net usage is a more autonomous, household-level practice.

Association between malaria history, treatment-seeking behavior variables, and IPTp uptake

The malaria RDT results were significantly associated with the uptake of IPTp. Specifically, women who tested positive for malaria were less likely to receive three or more doses of IPTp. The aOR for this association was 0.53 (95% CI = 0.31-0.92; p = 0.0227), indicating that women with a positive RDT result had a 47% lower likelihood of receiving the recommended IPTp regimen compared to those with a negative test result (Table 5).

**Table 5:** Association between malaria history, treatment-seeking behavior variables, and intermittent preventive treatment in pregnancy (IPTp) uptake cOR: crude odds ratios; aOR: adjusted odds ratio; *P-value <0.05

Variable	cOR (IC95%)	P-value	Global P-value	aOR (IC95%)	P-value	Global P-value
Had malaria in the past month						
Yes	1			-	-	-
No	0.93 (0.55-1.58)	0.7777	0.7777			
Sought consultation during the malaria episode						
No consultation	1		0.7005	-	-	-
Consultation at a conventional hospital	0.92 (0.41-1.99)	0.8378				
Consultation with a traditional healer	0.74 (0.31-1.69)	0.4801		-	-	-
Treatment received during the malaria episode						
Conventional treatment	1		0.1872	-	-	-
Traditional treatment	0.97 (0.53-1.76)	0.9101				
Both	1.81 (0.84-4.01)	0.1348		-	-	-
Medication purchase						
Prescription	1		0.0548	-	-	-
By self-medication	0.56 (0.26-1.18)	0.1337				
Both	1.10 (0.51-1.33)	0.8002		-	-	-
Rapid diagnostic test (RDT)						
Negative	1					
Positive	0.53 (0.31-0.92)	0.0227*	0.0227*	0.53 (0.31-0.92)	0.0227*	0.0227*

This finding may suggest that symptomatic malaria episodes, often associated with a positive RDT, could discourage routine ANC attendance or interrupt the IPTp schedule. Alternatively, a positive RDT result might reflect a missed opportunity for prevention, indicating gaps in early ANC engagement or irregular administration of IPTp before the onset of infection.

## Discussion

During the study, we observed that 62.55% of pregnant women had received at least three recommended doses of IPTp. This uptake rate is higher than the 51.3% national coverage reported in the 2023 annual report of the Cameroon NMCP [13]. However, it still falls short of the WHO’s target of 80% coverage, which is considered necessary to achieve substantial reductions in malaria-related morbidity during pregnancy [1].

While this higher rate may reflect encouraging progress in the study areas, it should be interpreted with caution. The sampling was limited to five health areas selected based on feasibility and security considerations, and the sample size was relatively small. Additionally, a potential urban or regional bias may have influenced the observed coverage, limiting the generalizability of these findings to the national level.

This situation suggests that, despite ongoing efforts to improve awareness and access to care, persistent barriers may be associated with suboptimal IPTp uptake among pregnant women, particularly in underserved or hard-to-reach areas.

The analysis of sociodemographic factors identified age as a significant determinant of IPTp uptake. Women aged between 25 and 30 years, as well as those over 30, had significantly higher odds of receiving the recommended three doses compared to younger women. This result aligns with findings from other studies conducted in sub-Saharan Africa, which report higher IPTp uptake among older pregnant women, possibly due to a greater perception of malaria-related risks during pregnancy and increased familiarity with health services [14,15].

In our sample, older women also tended to have more frequent ANC visits, which may partly explain their higher IPTp uptake. A possible explanation is that older women often have more pregnancy experience and are more likely to prioritize regular attendance at ANC. They may also benefit from stronger social and family support networks, which can facilitate timely access to maternal health services [16].

Marital status also positively influenced adherence to IPTp in our study. Married women and those living in cohabitation showed better adherence to the IPTp protocol compared to women in non-cohabiting relationships. This association may be explained by increased partner support, which plays a crucial role in decision-making related to maternal healthcare, including the use of preventive treatments [14]. Literature indeed shows that women benefiting from a stable and supportive environment, characterized by active partner support, are generally better informed and more inclined to adhere to recommended medical protocols, including IPTp [17,18]. It would therefore be beneficial to promote increased male partner involvement in antenatal consultations to further strengthen adherence to IPTp.

The frequency of ANC visits was identified as another significant determinant of adherence to IPTp. Participants who had more than two prenatal visits were more likely to receive the recommended doses of IPTp compared to those who had fewer visits. This result is consistent with reports from several other African countries where an increased number of prenatal visits is directly associated with better IPTp coverage [19,20]. These observations suggest that the regularity of prenatal visits is essential to ensure adequate IPTp coverage, as each additional visit increases opportunities for preventive treatment administration. Thus, it is crucial to encourage pregnant women to initiate ANC early and to continue regularly throughout pregnancy to maximize the effectiveness of malaria prevention [21].

Parity was also identified as a factor positively associated with optimal IPTp use in this study. Primiparous and multiparous women were significantly more likely to have received at least three doses of IPTp compared to nulliparous women. Similar results are reported in other contexts, indicating that prior pregnancy experience and increased awareness of malaria-related risks during pregnancy contribute to better adherence to preventive protocols [22,23]. These observations highlight the need to intensify education and awareness campaigns, specifically targeting first-time pregnant women, to ensure better understanding and early and consistent adoption of IPTp from the first pregnancy.

Concerning the level of knowledge about malaria and IPTp proved to be a crucial factor associated with better adherence. Women with good knowledge on these topics were more likely to achieve optimal IPTp coverage compared to those with limited or moderate knowledge. This relationship has been observed in several other studies, confirming that awareness and education on the benefits of IPT significantly improve compliance [24,25]. Therefore, strengthening community-based awareness and education strategies should be a priority to improve IPTp coverage in endemic regions such as Cameroon.

Finally, regarding the results of RDT, our study found that pregnant women who received three or more doses of SP were observed to have lower odds of testing positive for malaria compared to those who received fewer than three doses. This finding is consistent with previous research indicating that adequate administration of IPTp is associated with a reduced prevalence of malaria infection among pregnant women [26-28]. Indeed, adherence to the recommended IPTp doses significantly reduces the plasma parasitic load, thereby decreasing the probability of a positive RDT result. Moreover, this reduction in malaria infection risk helps lower complications associated with malaria during pregnancy, such as maternal anemia and low birth weight. Enhancing regular access to IPTp could therefore not only improve maternal health but also significantly reduce the risk of malaria infections detected through RDTs during antenatal consultations.

## Conclusions

This study highlights the potential impact of optimal IPTp-SP uptake, which is associated with improved maternal health outcomes and reduced malaria-related morbidity during pregnancy in Cameroon. While the observed uptake rate is higher than recent national estimates, it remains insufficient to achieve the WHO’s recommended threshold.

The findings demonstrate that sociodemographic factors such as age and marital status, reproductive history, ANC attendance, and knowledge levels are key determinants of IPTp adherence. Notably, adequate IPTp coverage was associated with lower malaria prevalence as measured by RDTs, reinforcing the protective effect of the intervention. Moving forward, public health strategies should prioritize early and frequent ANC attendance, enhance educational campaigns on malaria prevention, and encourage male partner involvement. Continued efforts to improve the availability and delivery of IPTp-SP within ANC services remain critical to achieving malaria prevention goals and safeguarding maternal and fetal health.
